# Utilization of Decision Tree Algorithms for Supporting the Prediction of Intensive Care Unit Admission of Myasthenia Gravis: A Machine Learning-Based Approach

**DOI:** 10.3390/jpm12010032

**Published:** 2022-01-02

**Authors:** Che-Cheng Chang, Jiann-Horng Yeh, Hou-Chang Chiu, Yen-Ming Chen, Mao-Jhen Jhou, Tzu-Chi Liu, Chi-Jie Lu

**Affiliations:** 1 Department of Neurology, Fu Jen Catholic University Hospital, Fu Jen Catholic University, New Taipei City 24352, Taiwan; changcc75@gmail.com (C.-C.C.); newtoloet@gmail.com (Y.-M.C.); 2 Ph.D. Program in Nutrition and Food Sciences, Human Ecology College, Fu Jen Catholic University, New Taipei City 242062, Taiwan; 3 School of Medicine, Fu Jen Catholic University, New Taipei City 24205, Taiwan; M001074@ms.skh.org.tw (J.-H.Y.); m001012.hc@gmail.com (H.-C.C.); 4 Department of Neurology, Shin Kong Wu Ho-Su Memorial Hospital, Taipei 11101, Taiwan; 5 Department of Neurology, Kaohsiung Medical University, Kaohsiung 80708, Taiwan; 6 Department of Neurology, Shuang-Ho Hospital, Taipei Medical University, New Taipei City 23561, Taiwan; 7 Graduate Institute of Business Administration, Fu Jen Catholic University, New Taipei City 242062, Taiwan; aaa73160@gmail.com; 8 Department of Business Administration, Fu Jen Catholic University, New Taipei City, 242062, Taiwan; ziggzagg19971224@gmail.com; 9 Artificial Intelligence Development Center, Fu Jen Catholic University, New Taipei City 242062, Taiwan; 10 Department of Information Management, Fu Jen Catholic University, New Taipei City 242062, Taiwan

**Keywords:** myasthenia gravis, machine learning, intensive care unit, decision tree, predication

## Abstract

Myasthenia gravis (MG), an acquired autoimmune-related neuromuscular disorder that causes muscle weakness, presents with varying severity, including myasthenic crisis (MC). Although MC can cause significant morbidity and mortality, specialized neuro-intensive care can produce a good long-term prognosis. Considering the outcomes of MG during hospitalization, it is critical to conduct risk assessments to predict the need for intensive care. Evidence and valid tools for the screening of critical patients with MG are lacking. We used three machine learning-based decision tree algorithms, including a classification and regression tree, C4.5, and C5.0, for predicting intensive care unit (ICU) admission of patients with MG. We included 228 MG patients admitted between 2015 and 2018. Among them, 88.2% were anti-acetylcholine receptors antibody positive and 4.7% were anti-muscle-specific kinase antibody positive. Twenty clinical variables were used as predictive variables. The C5.0 decision tree outperformed the other two decision tree and logistic regression models. The decision rules constructed by the best C5.0 model showed that the Myasthenia Gravis Foundation of America clinical classification at admission, thymoma history, azathioprine treatment history, disease duration, sex, and onset age were significant risk factors for the development of decision rules for ICU admission prediction. The developed machine learning-based decision tree can be a supportive tool for alerting clinicians regarding patients with MG who require intensive care, thereby improving the quality of care.

## 1. Introduction

Myasthenia gravis (MG) is an acquired autoimmune neuromuscular disorder presenting as muscle weakness in the eye, bulbar, limbs, and respiratory muscles that worsens with repeated muscle motion [1,2]. The pathogenesis of MG is caused by antibodies against post-synaptic proteins in the neuromuscular junction, the most common being the anti-acetylcholine receptor (AChR) antibody that accounts for 70% of patients with MG, and 15% of patients with thymoma [3]. Currently, the management of MG has been well-documented in recent years, and the survival rate of patients has improved [4,5,6]. However, the relapse rate and severity varies; approximately 38% of MG patients experience remission, and 10% are refractory to conventional rescue therapy, requiring repeated hospitalization or intensive care unit (ICU) admission, resulting in a decline in the quality of life and functional ability [7]. One of the most serious complications of MG is myasthenic crisis (MC), which presents with respiratory distress requiring ventilator support [8]. About 10%–20% patient with MG will experience at least one episode of MC who require ICU admission, and it is also the major cause of morbidity and mortality in MG [9,10]. A 10-year retrospective review of ICU admission in MG showed that more than 50% of patients had systemic infection during MC and 50% of patients with MC required ventilator support. The majority reasons for cause of death were respiratory failure and sepsis [11].

Although MC can cause significant morbidity and mortality, specialized neuro-intensive care, including respiratory monitor, nutrition support, non-invasive positive pressure ventilation, and intubation, can produce a good long-term prognosis in patients with severe MG symptoms [12,13,14]. The mortality rate of myasthenia crisis declined from 40% to 5% recently because of the novel medication development and the improvement intensive care techniques, especially related to ventilation management [15]. Specialized intensive care can result in better prognosis [16]. A retrospective study shows that the mortality rate is high (up to 30%) in places where intensive care resources are scarce [17]. Therefore, it is critical for better prognosis of patients with MG to determine the need admission to intensive care unit for close monitoring, and immediate access to resuscitation facilities as early as possible. Due to these reasons, many studies have attempted to explore the predictive factors for ICU admission in myasthenia crisis [18,19].

Autoimmune diseases, including MG, have chronic and fluctuating courses along with a complex pathophysiology. Prediction of outcomes and risk factors for autoimmune diseases is difficult due to the different phenotypes of clinical presentation. Current medical treatment integrates machine learning (ML) methods that play a critical role in personalized medicine by providing computers with the ability to learn from experience without rules specified by humans [20]. The basic principle of ML is predictive performance on unseen data that assists doctors in improving care quality and making precise decisions [21]. In addition, ML algorithms automatically learn useful data representations and process different types of input data. Thus, ML fills a gap in learning from clinical experience. It translates the knowledge gained into clinical evidence, with computers capable of predicting clinical outcomes, recognizing disease patterns, detecting disease features, and optimizing treatment strategies [22].

Considering the heterogeneity of myasthenia crises and the importance of intensive care to improve prognosis, it is critical to conduct risk assessments to predict the need for intensive care. Few studies had identified the risk factors of requirement of intensive care or intubation in patient with MG, including higher MG activities of daily living (MG-ADL) scores, initial symptom of bulbar weakness, infection, higher PCO2, and higher Myasthenia Gravis Foundation of America (MGFA) clinical classification [18,23]. However, there is still a lack of research constructing an effective prediction model for investigating the need for intensive care and ICU admission specific to MG and providing a valid tool for clinical factors screening and clinical practice. Our study aimed to construct an explainable predictive model to predict ICU admission in patients with MG; this predictive model was developed for clinical practice, based on ML decision tree methods, in order to screen clinical factors and decision rules for clinical practice. To our knowledge, there are no studies using ML-based decision tree techniques for building predictive models for ICU admission in patients with MG. Three ML-based decision tree techniques, including classification and regression tree (CART), C4.5, and C5.0, were used to construct explainable predictive models for identifying important clinical factors and for developing decision rules for ICU admission in MG patients during hospitalization.

## 2. Materials and Methods

### 2.1. Participant and Study Design

**Figure 1:**
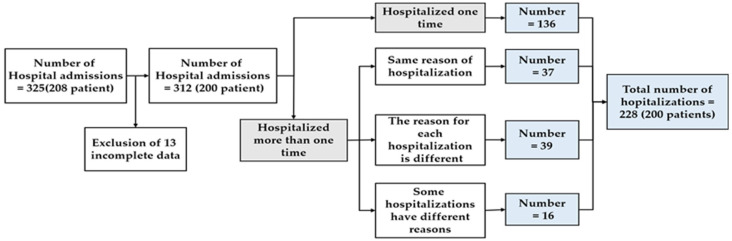
Subject identification process.

The data of 513 hospital admissions of patients with MG who were admitted to the Shin-Kong Wu Ho-Su Memorial Hospital in Taipei, Taiwan, between December 2015 and October 2018, were retrospectively analyzed. The inclusion criteria for patients with MG were (1) admission due to MG symptoms deterioration and (2) admission for MG-related management, including thymectomy or for immunotherapy. The exclusion criteria were (1) incomplete data and (2) admission that unrelated to myasthenia gravis. A total of 188 hospital admissions were excluded because they were not due to MG and 13 were excluded due to data loss, respectively. After cleaning the data, data from 200 patients with 312 hospital admissions were used for the analyses. The data in cases where the same patient had been hospitalized for the same reason were merged. Finally, a total number of 228 hospitalizations (including 200 patients) were used for the analysis (Figure 1). The protocol of this study was evaluated and deemed acceptable by the Research Ethics Review Committee of the Shin Kong Wu Ho-Su Memorial Hospital (No. 20190109R).

### 2.2. Data Collection and Clinical Measurement

A retrospective review of medical records, including information on the age, sex, age at diagnosis, disease duration, autoantibodies present, medications used, maximum dosage of corticosteroid before admission, thymic histology, history of thymectomy, treatment during hospitalization, and length of ICU admission, was conducted. Disease severity was graded according to the classification of the Myasthenia Gravis Foundation of America (MGFA) classification. Twenty clinical variables were collected. Table 1 lists twenty clinical factors (variables X1–X20) associated with patient with MG that may affect ICU admission.

The inclusion criteria for patients with MG were (1) Myasthenia Gravis Foundation of America (MGFA) class II and III, and (2) no medication adjustment in the last 6 months. The exclusion criteria were (1) unstable MG symptoms and (2) history of intensive immuno-modulation therapy, including immunoglobulins, high dose intravenous corticosteroid, or plasmapheresis, 6 months before enrollment—because the use of these short action immunotherapy means that the patient has a life-threatening phenomenon and unstable symptoms. Patients were eligible if they were diagnosed with MG based on the MGFA criteria. Briefly, the diagnosis of MG was based on fluctuating muscle weakness with fatigability, decreased symptom severity after use of acetylcholinesterase inhibitors, decremental changes in repetitive nerve stimuli on repetitive nerve stimulation test, or presence of anti-AchR autoantibodies.

The MGFA clinical classification was based on previous reviews that represented the clinical severity of the patient upon admission [24]. The maximum daily oral steroid dose before admission was recorded from the dosages during outpatient visits conducted within 1 month before hospitalization. Disease duration was defined as the time from the onset to the first visit after December 1, 2015.

The history of thymectomy was divided into three categories, as follows: (1) the patient had never undergone thymectomy; (2) the patient had undergone thymectomy during this admission; (3) thymectomy had been performed previously. The treatment during hospitalization included plasmapheresis, intravenous corticosteroid administration, intravenous immunoglobulin administration, and rituximab administration. Treatment with plasmapheresis was divided into three categories, as follows: (1) the patient did not undergo plasmapheresis; (2) the patient underwent five sessions; (3) the patient underwent more than five sessions. The serology status of MG autoantibodies included anti-AChR antibody and anti-MuSK antibody positivity or negativity, as well as double seronegativity.

As per the protocol of our hospital, patients with MG who are hospitalized for thymectomy must be observed in the ICU for 1 day after the operation. This distinguishes which groups of MG patients require ICU admission and then further divides them into two categories: ICU admission more than 1 day and less than 1 day. Most patients with MG are treated regularly in the ward, and those who require thymectomy are required for admission to the ICU for 1 day. Therefore, ICU admission in our study was defined as greater than 1 day.

In total, 228 patients included in the study (Figure 1) along with 20 clinical variables. Patient demographics are presented in Table 2. The average age at admission was 49.1 years with female predominance (61.4%). The average disease duration was 68.75 months. The average age at onset of MG symptoms was 43.2 years. Of the patients, 88.2% showed anti-AChR antibody positivity, 4.8% showed anti-MuSK antibody positivity, and 7.5% showed double seronegativity. A total of 12.7% of the patients were admitted to the ICU for more than 1 day.

The MGFA clinical classification at admission divided the patients into 5 groups: 24 patients (10.53%) were classified as class I, 88 patients (38.60%) as class II, 74 patients (32.46%) as class III, 26 patients (11.40%) as class IV, and 16 patients (7.02%) as having MG crisis. Regarding the medications used, 141 patients (60.1%) were treated with different oral immunosuppressants. According to the thymus histology, 110 patients (48.25%) had thymoma, and 67 patients (29.39%) had thymic hyperplasia. A total of 148 patients underwent thymectomy.

### 2.3. Machine Learning-Based Decision Tree Analysis

ML-based decision tree algorithms are popular and effective approaches for clinical/healthcare classification problems that visually represent the decision rules of generated predictions using a tree-shaped figure [25,26,27]. A decision tree is composed of nodes that are the optimum spilt of each feature, calculated by Gini or Entropy [28]. Gini measures the probability that any element of the dataset will be mislabeled when it is randomly labeled. Similar to Gini, Entropy measures information that indicates the disorder of the features with the target. Thus, this study utilized three most used tree-based algorithms including the following: CART, a decision tree based on the Gini method [29]; C4.5, a decision tree based on the Entropy method [30]; C5.0, an upgraded version of C4.5 that adds several facilities, such as variable misclassification costs [31]. The logistic regression (LR) is used as a baseline for performance comparison as it is a classic regression algorithm that focused on binary classification problems by calculating the natural logarithm of an odds ratio (logit). It predicted the logit of the dependent variable, that is, the ratio of probabilities of the dependent variable occurring from the logit of independent variables [32].

**Figure 2:**
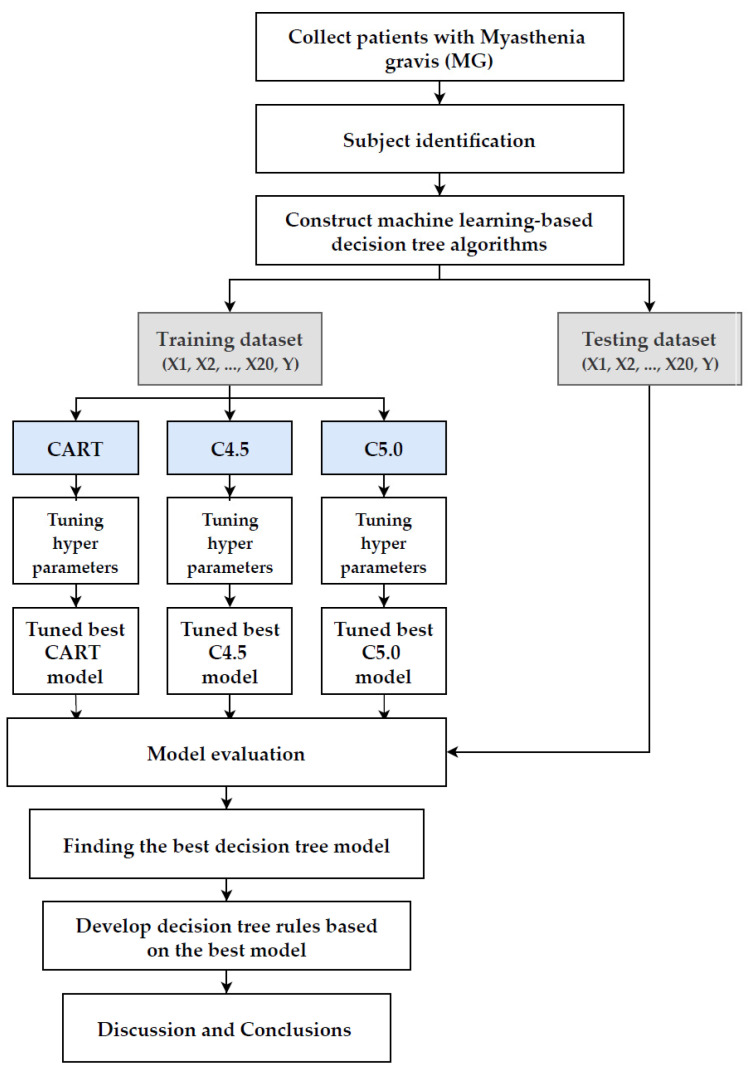
The overall flowchart of the proposed scheme.

Figure 2 shows the overall flowchart of the proposed scheme. In the proposed scheme, we first collected patients with MG and identified the subjects to prepare the dataset for model construction, then the dataset was randomly split into 80% training dataset for model building and 20% testing dataset for out of sample testing. Twenty clinical variables (X1 to X20) were used as predictor variables and ICU admission (Y) was applied as the target variable.

During the training phase, the hyperparameters of CART, C4.5, and C5.0 methods needed to be tuned for constructing a relatively well performed model, so we applied a 10-fold cross-validation technique for tuning the hyperparameters of each method. The training dataset was further randomly divided into 10 equal-sized folds, then 9 folds were used as a training dataset to build the model with a different set of hyperparameters, the remaining 1 fold was used as a validation dataset for model validation. The 10-fold cross-validation was repeated 10 times, by changing the fold of the validation dataset, thus the best hyperparameters set, which generated the best validation performance, based on the area under the receiver operating characteristic (ROC) curve (AUC) metric for each decision tree method, which was then used to construct the best CART, C4.5, and C5.0 models.

In the model evaluation phase, the testing dataset was used to evaluate the predictive performance of the best CART, C4.5, and C5.0 models. The metrics used for performance comparison were accuracy, sensitivity, specificity, AUC, and F1 score. In order to provide a more robust comparison, the process mentioned above was randomly repeated 10 times, and the averaged metrics were used to find the best decision model among the best CART, C4.5, and C5.0 models.

Finally, based on the best decision tree model, the decision rules could be developed, and the important clinical variables were identified. The rules were then discussed to improve the early recognition and care of patients with MG who need intensive care.

All methods were implemented in the R software with the 3.6.2 version. LR was constructed by the “blorr” package with the 0.3.0 version [33]; CART was constructed by the “rpart” package with the 4.1.15 version [34]; C4.5 was constructed by the “RWeka” package with the 0.4.42 version [35]; C5.0 was constructed by the “C50” package with the 0.1.5 version [36]. The hyperparameters of all the methods used were tuned by the “caret” package with the 6.0.88 version [37].

## 3. Results

**Figure 3:**
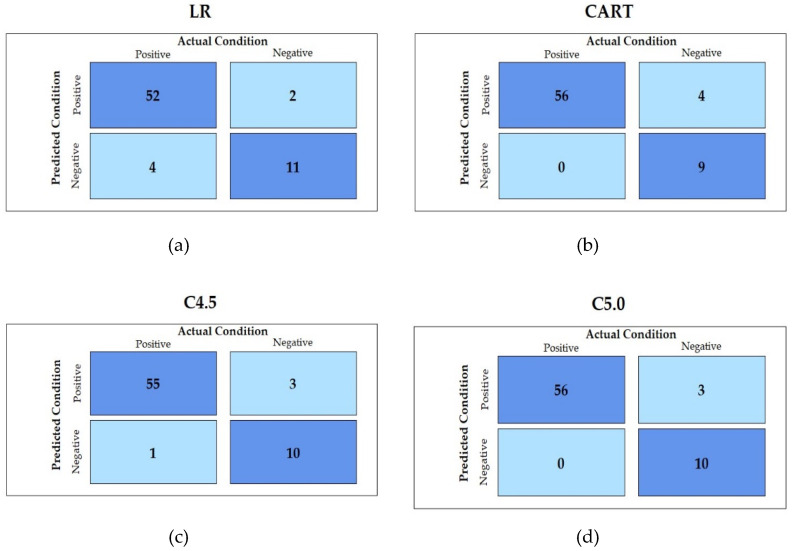
Confusion matrix of each method based on its best model: (**a**) LR; (**b**) CART; (**c**) C4.5; (**d**) C5.0.

According to the proposed scheme, for modeling effective CART, C4.5, and C5.0 models, the hyperparameters of each method were tuned and evaluated. The LR method without hyperparameters tuning—the baseline method—was constructed by using the proposed scheme. The values of the hyperparameters which generated the best CART, C4.5, and C5.0 models with the highest AUC values are listed in Table 3. Figure 3 uses confusion matrices to demonstrate the predicted results of LR, CART, C4.5, and C5.0 methods. From this figure, it can be observed that the best C5.0 method generated the best positive and negative predicted results compared with that of the best LR, CART, and C4.5 methods.

**Figure 4:**
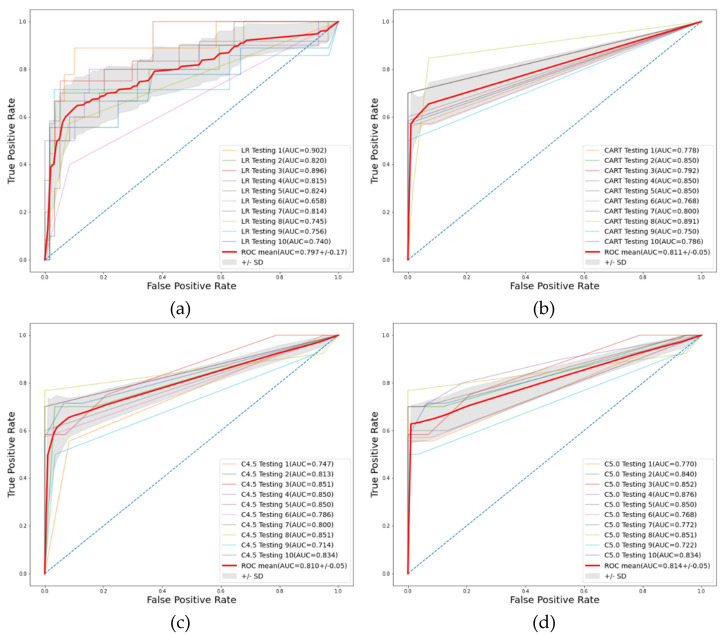
ROC curves of the four decision tree algorithms: (**a**) LR; (**b**) CART; (**c**) C4.5; (**d**) C5.0.

The performance of the LR, CART, C4.5, and C5.0 methods with 10 repetitions is shown in Table 4, with the average and standard deviation (SD) of the 5 metrics used in this study. As shown in the table, the CART, C4.5, and C5.0 methods have better AUC performance than the classic LR. Among all three decision tree algorithms, C5.0 had the highest average AUC (0.814), followed by CART and C4.5. The C5.0 method also performed best in terms of the accuracy (0.942), sensitivity (0.994), and F1 score (0.967). The ROC curves, as well as the one SD of the mean AUCs of all methods, are shown in Figure 4**.** The figure shows that C5.0 is the best predictive model in this study.

As C5.0 has the best AUC and outperforms the four competing methods, the important clinical factors and decision rules generated and suggested by the best C5.0 model are discussed.

## 4. Discussion

**Figure 5:**
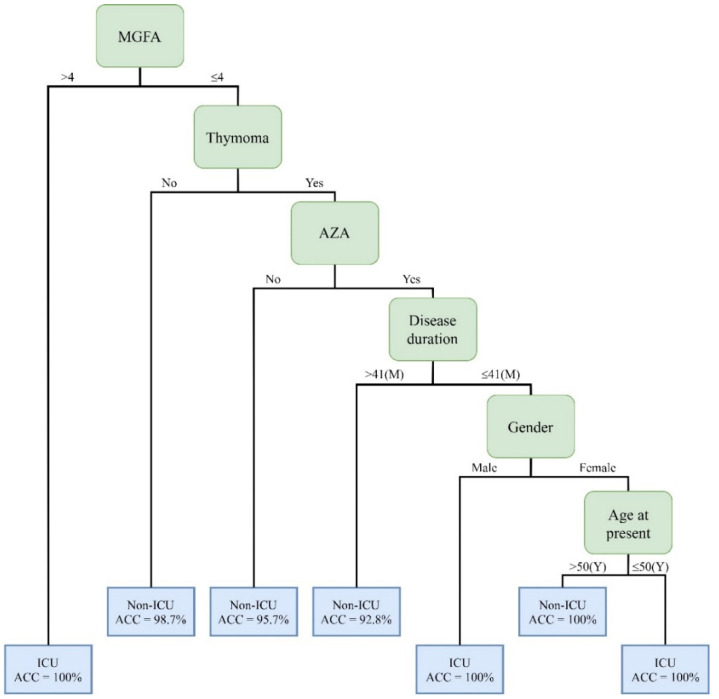
Decision rules for the prediction of ICU admission in MG patients based on important clinical factors of the best C5.0 model.

This is the first study to use the ML decision tree method for predicting ICU admission in patients with MG. The C5.0 method generated the best and most promising classification results and provided an output of six clinical features that were critical for determining the risk of ICU admission. Figure 5 shows the decision rules for the prediction of ICU admission in MG patients based on the six important clinical factors of the best model—the C5.0 model. Table 5 summaries decision rules of combinations of clinical factors from Figure 5. The rules in Figure 4 and Table 5 are then discussed to improve the early recognition and care of patients with MG who need intensive care.

The MGFA classification and the presence of thymoma were two of the important physiological indices. The MGFA clinical classifications are used to identify the different clinical features and severity of patients with MG [38]. The higher the class of MGFA, the more severe the symptoms. The severity at the onset of MG constituted a grave risk in our patients, and the MGFA class at admission reflected the severity of MG upon admission. The association of disease severity with a high risk of death could be explained by the frequent involvement of the bulbar and respiratory muscles in these individuals. An MGFA score of 4 indicates severe MG symptoms or an acute crisis that may require ventilator support or intensive care [38]. Another physiological index for MG that influences ICU admission is the presence of thymoma, which was found in approximately 15%–60% of MG cases [39,40,41]. The presentation of thymoma is caused by an immune response in thymoma cells [42]. Studies showed controversial outcomes and disease severities regarding different thymic pathologies [43]. Zhang et al. also demonstrated that MG patients with thymoma had a poorer prognosis than patients without thymoma [39] due to serious disease manifestations. Our results are in line with those of previous studies that show the important role of thymoma in ICU admission among patients with MG.

In addition, this study demonstrated that some treatments have influenced ICU admission in patients with MG, including treatment with oral azathioprine (AZA). According to the international guidelines for the management of MG, immunosuppressive therapy is used in patients with MG who have poor response to pyridostigmine alone [5]. AZA was added early if the patient had comorbidities, such as diabetes, significant depression (with steroids potentially exacerbating their mood), osteoporosis, and leg ulcerations and could not tolerate steroid treatment [39,44]. Therefore, patients used AZA due to comorbidities and side effects that cause physicians to change their medications. This also meant that such patients are likely to have other comorbid diseases that influence their need for ICU admission.

The onset age and disease duration also influenced ICU admission in patients with MG. Our results showed that late-onset MG, defined as MG at an onset age >50 years, had a negative prediction for ICU admission. Previous studies indicated that patients with late-onset MG are likely to have a thymoma and a severe disease that is difficult to treat [45,46]. An observational cross-sectional multicenter study showed that patients with late-onset MG may present with more severe symptoms than younger patients [47]. Other large cohort studies suggested that late-onset MG patients are prone to increased disease severity, and the mortality rate increased in the elderly [15,48]. This is because elderly patients with MG tend to have comorbidities and complications, such as sepsis, resulting in long hospital stays and high costs [49]. Old onset age of MG could be associated with an increased susceptibility to autoimmune diseases, due to immune dysregulation and increased inflammatory background that can cause a high production of autoantibodies [49]. Although the elderly have a more severe presentation than younger patients, they require a low dosage of medications, have better prognosis to management, and a short weaning time in the ICU after a myasthenic crisis [48]. This may explain why elderly patients were less likely to be admitted in ICU in our study.

Our findings found that disease duration shorter than 41 months was a factor that could influence ICU admission in patients with MG [50]. Many reports have not concluded that disease duration is closely associated with the prognosis in patients with MG [51]. A large retrospective study, that is very similar to ours, demonstrated that the majority of deaths occurred 5–10 years after the onset of the disease and rarely within 5 years; however, the risk of death tended to decrease after 15 years of the prevalence of the disease [52]. Since it is an autoimmune disease, proper medical intervention helps stabilize the symptoms significantly [52]. This may be because the longer the course of the disease, the more stable the drug treatment and the better the psychological adaptation of the patient to the disease, resulting in a lower the rate of hospitalization requiring ICU admission. Our findings, distinguished from other studies, defined an absolute point—that disease duration of 41 months can predict ICU admission. To the best of our knowledge, no previous study has defined how long the duration of the disease may affect the outcomes of MG, and our studies provide new insights into the clinical intensive care of MG.

Management of MG upon admission would benefit from a good understanding of the disease course. In this study, many of the risk factors associated with the prognosis of hospitalized MG patients requiring ICU admission were identified using the C5.0 algorithm. C5.0 is a promising ML-based decision tree algorithm and has been successfully used in clinical/healthcare issues [26,53]. Previously published predictive factors of the prognosis of MG in MG deterioration, severity, and hospital stay have used multivariable logistic regression analyses [49,54,55]. This research found that, compared with LR, C5.0 and the other two ML-based decision tree algorithms can generate a better accuracy. The results confirmed that ML algorithms are important for disease detection and risk assessment in several autoimmune diseases, such as rheumatoid arthritis [56,57]. ML can help clinicians detect and process clinically useful information in small patient samples, gain a good understanding of disease courses, adapt treatments earlier, and find the best management plan [56]. Most importantly, the natural course of an MG phenotype is highly variable and impacts the disease clinical course and prognosis significantly, with no good prediction target [58]. Our study tried to use the ensemble learning method to construct a decision tree model for predicting ICU admission in MG patients during hospitalization for future studies.

Some investigators have evaluated the potential risk factors of ICU care in patient with MG. A retrospective study showed that higher MG-ADL scores with bulbar involvement and higher MGFA classification were associated more frequently in the ICU group. Better outcomes may be obtained with early intensive care management if the patient presents with those factors [18]. Liu et al. suggested that CO2 level before intubation and the score on MG-ADL at onset may be associated with prolonged ICU stays [20]. Some respiratory management had been found for prevention of intubation, and prolonged ventilator use in patients with MG had also been reported, including bilevel positive airway pressure (BiPAP) [13], and hypercapnia could also be a predicting factor for early intubation under BiPAP [12]. However, there was still no valid guidance that could identify the need for intensive care in MG early.

This study had several clinical implications. First, the variable phenotype of MG makes it difficult to determine the optimal management plan and prognosis; therefore, physicians can use this decision tree model to identify patients likely to have ICU admission during hospitalization. Second, this study may provide a possible tool for clinical guidance for ICU clinicians to improve intensive care quality. This is because patients with MG who are likely to stay in the ICU have a high mortality, require a ventilator, and suffer severe disability. Due to the improvement in management and development of intensive care techniques, the fatality rate of MG has been less than 5% in the recent years [15]. An early diagnosis of MG patients who need ICU can improve survival and outcomes. Third, as ML expands its access to health care for patients with rheumatic disease, this study, using ML methods to assess ICU admission, revealed a new point of view, and the results showed that ML methods can provide a predictive accuracy. Using this method could improve the quality of care of patients with MG. The model can be combined with other clinical parameters, including the respiratory rate, difficulty with phonation, weak neck muscles, oxygenation, and advanced electronic functionalities, such as monitoring of partial pressure of carbon dioxide and testing of vital capacity. This study recommends the model as a primary benchmarking tool to be used in the evaluation of MG patients during hospitalization.

There are some limitations to this study. First, the usefulness of the model is probably restricted to our hospital because of inter-hospital differences that impact the model of ICU strategy in MG. Second, the data were acquired from chart reviews, and the specific details of each patient, such as the quantitative MG score, which can quantify the severity and outcomes of treatment, were not available for analysis. Third, due to the heterogeneity of MG symptoms, our model failed to account for additional clinical factors known to influence ICU admission. These include the partial pressure of carbon dioxide at admission, the activities of daily living score at myasthenia crisis onset, and nosocomial infection [18,20]. Finally, these models were chosen based on clinical data. Other variables, such as corticosteroid dose, treatment period, and previous underlying diseases, including chronic obstructive pulmonary disease and diabetes, were not included in our analysis. Multicenter studies should be performed to ensure that the results are not due to artifacts in ML systems. In addition, multicenter studies may complete the framework of this study.

## 5. Conclusions

This study uses an ML-based decision tree approach to predict ICU admission in patients with MG. Decision tree methods are promising tools that can build a predictive model of ICU admission in patients with MG, and they can provide physicians with information to evaluate the potential risk of ICU admission in patients with MG. Due to the varying clinical presentation of patients with MG, the model produced can be used for performance benchmarking and as a supportive tool for alerting clinicians regarding the patients with MG who require intensive care and management; therefore, enabling clinicians to provide timely and effective treatment, improving care quality and patient outcomes.

## Figures and Tables

**Table 1:** Variable definitions.

	Variables	Description	Unit
X1	Age at admission	Age of first visit after 1 December 2015	Years
X2	Disease duration	Time from the onset to the first visit after 1 December 2015	Months
X3	Age at onset	Age of MG symptoms onset	Years
X4	Gender	Male/Female	—
X5	MGFA clinical classification	The maximum MGFA clinical severity during enrollment period: 1: Class I: ocular muscle weakness 2: Class II: Mild limbs, axial, bulbar or respiratory weakness 3: Class III: Moderate limbs, axial, bulbar or respiratory weakness 4: Class IV: Severe limbs, axial, bulbar or respiratory weakness 5: Class V: Intubation	—
X6	Thymoma	Thymus present with thymoma	Yes/No
X7	Hyperplasia	Thymus present with thymic hyperplasia	Yes/No
X8	Thymectomy	History of received thymectomy 0: No 1: Received thymectomy at presented hospitalization 2: Received thymectomy before	—
X9	Anti-AChR Ab	Serology of autoantibody against Anti-AChR	Yes/No
X10	Anti-MuSK Ab	Serology of autoantibody against Anti-MuSK Ab	Yes/No
X11	dSN	Double seronegative	Yes/No
X12	PSL Maximum daily dose	The maximum dose of corticosteroid from the first visit between December 2015 and October 2018	mg
X13	OI	Treatment with Oral Immunosuppressant during enrollment period	Yes/No
X14	AZA	Treatment with Azathioprine during enrollment period	Yes/No
X15	MMF	Treatment with Mycophenolate mofetil during enrollment period	Yes/No
X16	OT	Treatment with Oral Tacrolimus during enrollment period	Yes/No
X17	IVIG	Treatment with Intravenous immunoglobins during enrollment period	Yes/No
X18	PP	Treatment with plasmapheresis during enrollment period 1: No 2: 5 sessions 3: >5 sessions	—
X19	IC	Treatment with intravenous corticosteroid during enrollment period	Yes/No
X20	RTX	Treatment with Rituximab during enrollment period	Yes/No
Y	ICU admission	ICU admission was defined as greater than 1 day 0: ≤1 day 1: >1 day	—

Note: Anti-AChR Ab—anti-acetylcholine receptor; Anti-MuSK Ab—muscle-specific receptor tyrosine kinase; dSN—double-seronegative; PSL—prednisolone; OI—oral immunosuppressant; AZA—azathioprine; MMF—mycophenolate mofetil; IVIG—intravenous immunoglobins; PP—plasmapheresis; IC—intravenous corticosteroid; RTX—rituximab; OT—oral tacrolimus; ICU—intensive care unit.

**Table 2:** Subject demographics.

Characteristics		Metrics
Basic Information:		Mean ± SD
X1:	Age at admission	49.14 ± 17.01
X2:	Disease duration	68.75 ± 84.40
X3:	Age at onset	43.22 ± 17.43
X4:	Gender:	N (%)
	Male	88(38.60%)
	Female	140(61.40%)
X5:	MGFA clinical classification:	N (%)
	Class I	24(10.53%)
	Class II	88(38.60%)
	Class III	74(32.46%)
	Class IV	26(11.40%)
	Class V	16(7.02%)
Thymus:		N (%)
X6:	Thymoma:	
	No	118(51.75%)
	Yes	110(48.25%)
X7:	Hyperplasia:	
	No	161(70.61%)
	Yes	67(29.39%)
X8:	Thymectomy:	
	No	80(35.09%)
	Received thymectomy at presented	93(40.79%)
	Received thymectomy before	55(24.12%)
Autoantibody:		N (%)
X9:	Anti-AChR Ab:	
	No	27(11.84%)
	Yes	201(88.16%)
X10:	Anti-MuSK Ab:	
	No	217(95.18%)
	Yes	11(4.82%)
X11:	dSN:	
	No	211(92.54%)
	Yes	17(7.46%)
Treatment status:		Mean ± SD
X12:	PSL Maximum daily dose	14.60 ± 15.68
X13:	OI:	N (%)
	No	91(39.91%)
	Yes	137(60.09%)
X14:	AZA:	N (%)
	No	152(66.67%)
	Yes	76(33.33%)
X15:	MMF:	N (%)
	No	219(96.05%)
	Yes	9(3.95%)
X16:	OT:	N (%)
	No	222(97.37%)
	Yes	6(2.63%)
X17:	IVIG:	N (%)
	No	213(93.42%)
	Yes	15(6.58%)
X18:	PP:	N (%)
	No	66(28.95%)
	5 sessions	131(57.46%)
	>5 sessions	31(13.60%)
X19:	IC:	N (%)
	No	185(81.14%)
	Yes	43(18.86%)
X20:	RTX:	N (%)
	No	222(97.37%)
	Yes	6(2.63%)
Y:	ICU admission:	N (%)
	≤1 day	199(87.28%)
	>1 day	29(12.72%)

Note: Anti-AChR Ab—anti-acetylcholine receptor; Anti-MuSK Ab—muscle-specific receptor tyrosine kinase; dSN—double-seronegative; PSL—prednisolone; OI—oral immunosuppressant; AZA—azathioprine; MMF—mycophenolate mofetil; IVIG—intravenous immunoglobins; PP—plasmapheresis; IC—intravenous corticosteroid; RTX—rituximab; OT—oral tacrolimus; ICU—intensive care unit.

**Table 3:** Summary of the values of the hyperparameters for the best CART, C4.5, and C5.0 models.

Methods	Hyperparameters	Value	Meaning
CART	minispilt	20	The minimum number of observations that must exist in a node for a split to be attempted.
	minibucket	20	The minimum number of observations in any terminal node.
	maxdepth	10	The maximum depth of any node of the final tree.
	xval	10	Number of cross-validations.
	cp	0.0781	Complexity parameter: The minimum improvement in the model needed at each node.
C4.5	C	0.5	The confidence threshold tree size of pruning.
	M	3	The minimum number of instances per leaf.
C5.0	trials	20	The number of boosting iterations.
	model	Tree	The model growing of type.
	winnow	F	The tree be decomposed into a rule-based model.

CART—classification and regression tree; C4.5—C4.5 decision tree; C5.0—C5.0 decision tree; cp—complexity parameter.

**Table 4:** The performance of the LR, CART, C4.5, and C5.0 methods.

Methods	Accuracy Mean (SD)	Sensitivity Mean (SD)	Specificity Mean (SD)	AUC Mean (SD)	F1 Score Mean (SD)
LR	0.862(0.08)	0.892(0.11)	0.702(0.27)	0.797(0.17)	0.915(0.06)
CART	0.942(0.02)	0.993(0.02)	0.633(0.10)	0.811(0.05)	0.967(0.01)
C4.5	0.929(0.03)	0.978(0.03)	0.639(0.09)	0.810(0.05)	0.959(0.02)
C5.0	0.942(0.02)	0.994(0.02)	0.639(0.09)	0.814(0.05)	0.967(0.01)

LR—logistic regression; CART—classification and regression tree; C4.5—C4.5 decision tree; C5.0—C5.0 decision tree.

**Table 5:** Summarized decision rules of combinations of clinical factors.

Rules No.	Combinations of Clinical Factors	Cases	Positive/Negative	Accuracy
1	MGFA (>4)	9	Positive	100%
2	MGFA (≤4) + Thymoma (No)	81	Negative	98.7%
3	MGFA (≤4) + Thymoma (Yes) + AZA(No)	47	Negative	95.7%
4	MGFA (≤4) + Thymoma (Yes) + AZA(Yes) + Disease duration (>41)	14	Negative	92.8%
5	MGFA (≤4) + Thymoma (Yes) + AZA(Yes) + Disease duration (≤41) + Gender (Male)	4	Positive	100%
6	MGFA (≤4) + Thymoma (Yes) + AZA(Yes) + Disease duration (≤41) + Gender (Female)+Age at present (≤50)	2	Positive	100%
7	MGFA (≤4) + Thymoma (Yes) + AZA(Yes) + Disease duration (≤41 + Gender (Female)+Age at present (>50)	2	Negative	100%

Note: AZA—Azathioprine.

## Data Availability

Data available on request due to privacy/ethical restrictions.
